# Hypomagnesemia: An Overlooked Cause of Delirium and Cardiac Complications in an Elderly Patient

**DOI:** 10.7759/cureus.92427

**Published:** 2025-09-16

**Authors:** Zambu Kyaw, Khaing Cherry Phyo, Thyn Thyn

**Affiliations:** 1 Acute Medicine Department, Barnsley Hospital NHS Foundation Trust, Barnsley, GBR; 2 Emergency Department, Barnsley Hospital NHS Foundation Trust, Barnsley, GBR; 3 Geriatrics Department, Barnsley Hospital NHS Foundation Trust, Barnsley, GBR

**Keywords:** delirium, elderly, electrolyte imbalance, frailty, hypomagnesemia, myocardial injury

## Abstract

Delirium is a frequent complication in elderly patients and is often multifactorial, with electrolyte imbalances representing a major contributor. While sodium, potassium, and calcium disorders are commonly evaluated, hypomagnesemia is an overlooked but clinically significant cause of both neurological and cardiac manifestations. Magnesium is essential for neuronal stability, neurotransmitter regulation, and calcium channel modulation. Its deficiency can lead to neuropsychiatric symptoms, arrhythmias, and myocardial injury. We report the case of an 81-year-old woman who presented with acute confusion following several days of diarrhea. Investigations revealed severe hypomagnesemia (serum magnesium: 0.3 mmol/L), ST-segment depression, prolonged QT interval, and elevated troponin levels. She was managed as a type 2 myocardial infarction in the context of sepsis and electrolyte imbalance. Prompt intravenous magnesium replacement resulted in rapid resolution of delirium and cognitive improvement. This case underscores the importance of routine assessment of magnesium levels in elderly patients presenting with delirium, especially in the presence of gastrointestinal losses or cardiovascular comorbidities, and highlights the reversibility of neurological and cardiac complications when deficiency is corrected.

## Introduction

Delirium is an acute, fluctuating syndrome of encephalopathy causing disturbed consciousness, attention, cognition, and perception [1]. It is a common and often multifactorial syndrome in older adults, associated with increased morbidity, prolonged hospitalization, and poor outcomes [1,2]. The pathophysiology is complex, but delirium frequently arises from acute medical conditions such as infection, systemic inflammation, metabolic derangements, or electrolyte imbalance. While sodium, potassium, and calcium abnormalities are routinely evaluated, magnesium deficiency is frequently overlooked despite its profound neurological and cardiovascular effects [3,4].

Magnesium is the second most abundant intracellular cation and plays a critical role in neuronal excitability, neurotransmitter release, synaptic plasticity, and ion channel modulation [4]. Deficiency may result in a wide spectrum of neurological and cardiovascular manifestations, including confusion, seizures, arrhythmias, and myocardial injury. Recent evidence has shown that hypomagnesemia is independently associated with incident delirium in hospitalized older persons, suggesting it may be an under-recognized but clinically relevant risk factor [5]. The interface between dementia and delirium adds diagnostic complexity, as acute cognitive fluctuations may be misattributed to the progression of baseline cognitive impairment [6].

Magnesium deficiency is common in older adults, often due to poor intake, diarrheal loss, renal wasting, or the effect of medications such as diuretics and proton pump inhibitors [3,4]. Despite its prevalence, magnesium is less frequently checked in standard panels, leading to missed diagnoses. Given its reversible nature, early recognition and treatment of hypomagnesemia can significantly improve outcomes in patients presenting with delirium and cardiovascular complications.

We present the case of an elderly woman who developed delirium, sepsis, and myocardial injury in the context of profound hypomagnesemia, highlighting the importance of early recognition and correction of this reversible electrolyte disturbance.

## Case presentation

An 81-year-old woman with a background of prior myocardial infarction with coronary artery bypass grafting, treated breast cancer, and known frailty was brought to the emergency department with acute confusion and general malaise. Her family reported several days of diarrhea, poor oral intake, and increasing frailty. She also developed a fever and urinary symptoms. There was no history of chest pain, palpitations, shortness of breath, or focal neurological deficits.

On arrival, she was hemodynamically stable and afebrile, with a National Early Warning Score (NEWS) of 0: blood pressure, 135/80 mmHg; heart rate, 88 beats per minute; respiratory rate, 14 breaths per minute; oxygen saturation, 98% on room air; and temperature 36.5°C. Physical examination revealed no focal neurological signs, but she remained disoriented. Initial laboratory investigation findings are summarized in Table 1.

**Table 1:** Summary of initial laboratory investigations.

Parameters	Patient values	Reference range
Urinalysis: protein	2+	Negative
Urinalysis: nitrate	2+	Negative
Urinalysis: leucocyte	Positive	Negative
Urinalysis blood	2+	Negative
Serum sodium	133 mmol/L	133–146 mmol/L
Serum potassium	3.4 mmol/L	3.5–5.3 mmol/L
Serum creatinine	104 µmol/L	44–71 µmol/L
Serum magnesium	0.3 mmol/L	0.7–1.0 mmol/L
C-reactive protein	184 mg/L	<5 mg/L
White cell count	11 × 10^9^/L	4–11 × 10^9^/L
High-sensitivity troponin I	2,039 → 1,036 ng/L	<14 ng/L
Vitamin D	14.8 nmol/L	>50 nmol/L
Urine culture	>10^5^ CFU/mL Escherichia coli	No growth

**Figure 1:**
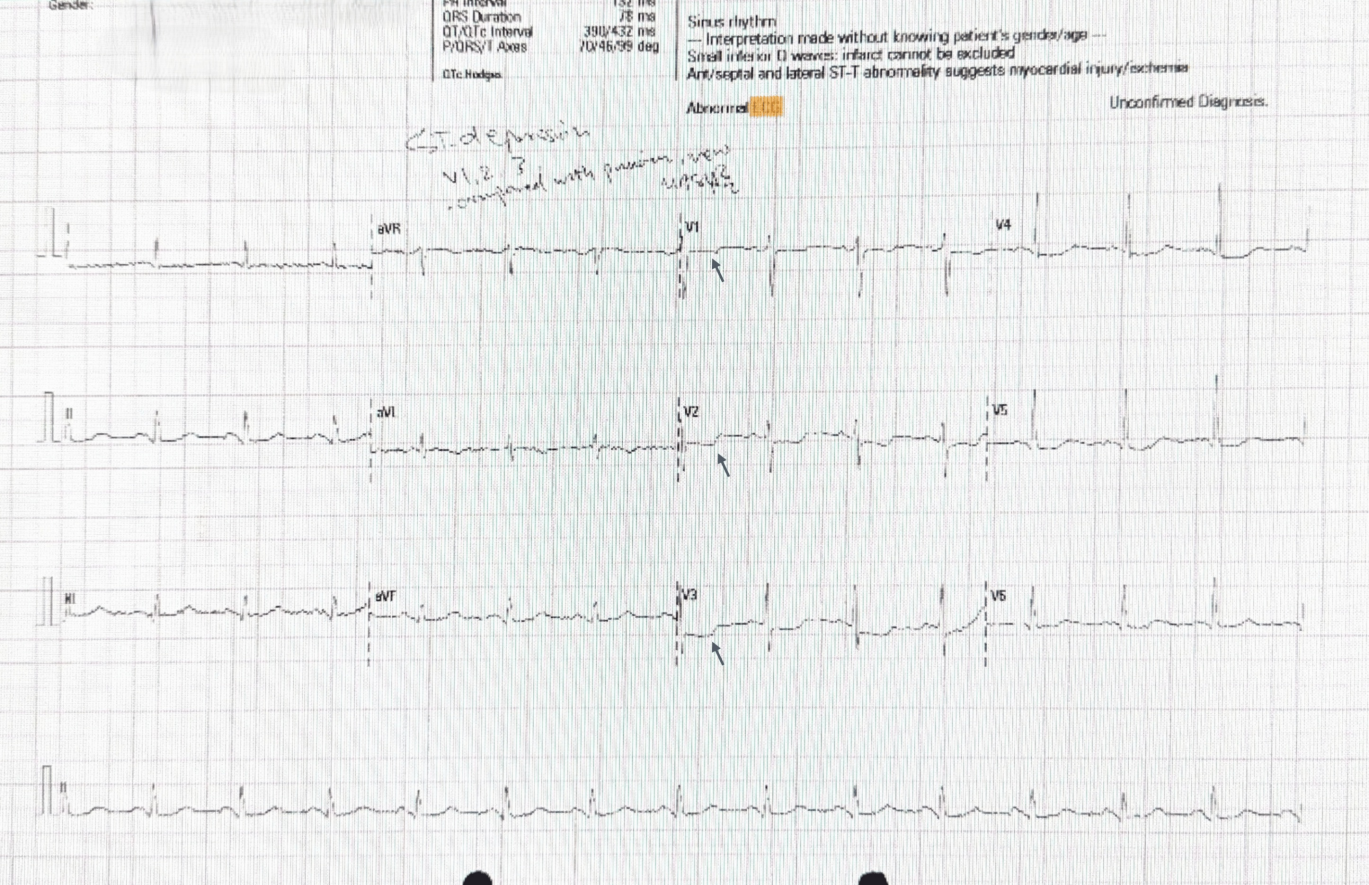
ECG showing ST-segment depression in V1-V3, indicated by arrows.

ECG showed new ST-segment depression in V1-V3 (Figure 1).

**Figure 2:**
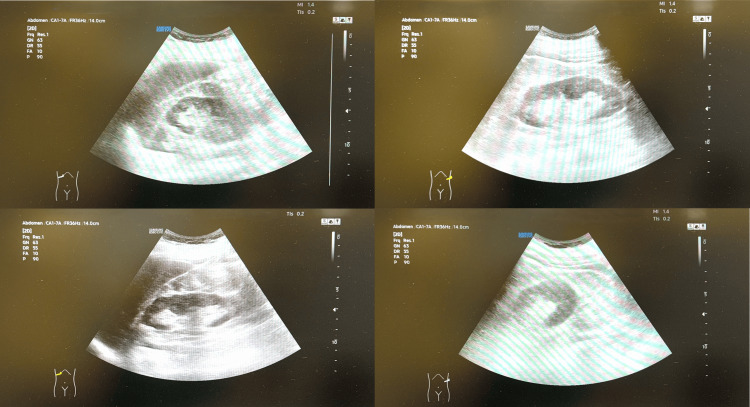
Ultrasound of the urinary tract showing normal kidneys.

On CT of the head, no acute intracranial hemorrhage, surface collection, infarct, or mass effect was detected. On renal ultrasound, both kidneys appeared normal, and the urinary bladder was underfilled (Figure 2).

The working diagnoses were urosepsis, severe hypomagnesemia, delirium, and myocardial injury. She was commenced on intravenous antibiotics, intravenous magnesium replacement, and dual antiplatelet therapy for presumed acute coronary syndrome. Following a cardiology review, the troponin rise was attributed to type 2 myocardial infarction (demand ischemia) secondary to infection and electrolyte disturbance, and antiplatelet therapy was de-escalated to a single agent. A loading dose of vitamin D supplementation was initiated.

Intravenous magnesium sulfate infusion (20 mmol in 250 mL of 0.9% saline) led to a prompt rise in serum magnesium (0.7 mmol/L) and rapid improvement in her confusion. Over the following days, her cognition returned to baseline, and her electrolytes and inflammatory markers normalized. A careful follow-up plan was arranged to monitor her clinical status, electrolyte balance, and vitamin D levels. She was subsequently discharged with outpatient follow-up in cardiology, frailty, and memory services.

## Discussion

This case illustrates the under-recognized role of hypomagnesemia in precipitating delirium and myocardial injury in elderly patients. Electrolyte disturbances, particularly hypomagnesemia, are increasingly recognized as modifiable risk factors for delirium [5,7]. In hospitalized older adults, low magnesium levels have been independently associated with incident delirium [5].

Magnesium deficiency is often multifactorial, commonly resulting from gastrointestinal losses, renal wasting, or medications such as diuretics and proton pump inhibitors [3,4]. In this patient, several days of diarrhea likely contributed to severe depletion. Neurologically, magnesium deficiency disrupts synaptic transmission, increases neuronal excitability, and manifests as confusion, agitation, or seizures [4].

From a cardiovascular perspective, magnesium deficiency prolongs repolarization, predisposes to arrhythmias, and may exacerbate myocardial ischemia [8,9]. Observational studies have linked low serum magnesium with adverse electrocardiographic changes, increased mortality, and coronary artery disease risk [8-11]. In this patient, profound hypomagnesemia contributed to type 2 myocardial injury in the setting of sepsis.

Vitamin D deficiency may have also contributed to delirium in this case. Evidence suggests that progressively lower vitamin D levels are associated with an increased risk of incident hospital-diagnosed delirium [12]. This underscores the importance of monitoring both vitamin D status and electrolyte balance in older patients presenting with acute cognitive changes.

Importantly, correction of hypomagnesemia leads to rapid improvement, as seen here. Clinicians should be aware that hypokalemia and hypocalcemia may be refractory to correction until magnesium is replaced [4].

Despite its clinical importance, magnesium disturbance is frequently underappreciated as a potential contributor to delirium, particularly in frail older patients with multiple comorbidities. Its contribution to both cognitive disturbance and cardiovascular instability reinforces the need for routine assessment. Broader awareness among clinicians could support earlier intervention and improved patient outcomes.

## Conclusions

Hypomagnesemia is a reversible but frequently overlooked cause of delirium and myocardial injury in elderly patients. This case highlights the unique presentation of severe hypomagnesemia with concurrent neuropsychiatric symptoms and cardiac abnormalities. Early recognition and correction can prevent complications, restore baseline cognitive function, and reduce cardiovascular risk. Magnesium assessment should be included in the routine evaluation of elderly patients presenting with delirium, especially in the presence of gastrointestinal disturbances, frailty, or cardiac comorbidities.
